# High-Speed Video-Oculography for Measuring Three-Dimensional Rotation Vectors of Eye Movements in Mice

**DOI:** 10.1371/journal.pone.0152307

**Published:** 2016-03-29

**Authors:** Takao Imai, Yasumitsu Takimoto, Noriaki Takeda, Atsuhiko Uno, Hidenori Inohara, Shoichi Shimada

**Affiliations:** 1 Department of Otorhinolaryngology - Head and Neck Surgery, Osaka University Graduate School of Medicine, Osaka, Japan; 2 Department of Otolaryngology, Suita Municipal Hospital, Osaka, Japan; 3 Department of Otorhinolaryngology - Head and Neck Surgery, Tokushima University Graduate School of Medicine, Tokushima, Japan; 4 Department of Otorhinolaryngology - Head and Neck Surgery, Osaka General Medical Center, Osaka, Japan; 5 Department of Neuroscience and Cell Biology, Osaka University Graduate School of Medicine, Osaka, Japan; University of Iowa, UNITED STATES

## Abstract

**Background:**

The mouse is the most commonly used animal model in biomedical research because of recent advances in molecular genetic techniques. Studies related to eye movement in mice are common in fields such as ophthalmology relating to vision, neuro-otology relating to the vestibulo-ocular reflex (VOR), neurology relating to the cerebellum’s role in movement, and psychology relating to attention. Recording eye movements in mice, however, is technically difficult.

**Methods:**

We developed a new algorithm for analyzing the three-dimensional (3D) rotation vector of eye movement in mice using high-speed video-oculography (VOG). The algorithm made it possible to analyze the gain and phase of VOR using the eye’s angular velocity around the axis of eye rotation.

**Results:**

When mice were rotated at 0.5 Hz and 2.5 Hz around the earth’s vertical axis with their heads in a 30° nose-down position, the vertical components of their left eye movements were in phase with the horizontal components. The VOR gain was 0.42 at 0.5 Hz and 0.74 at 2.5 Hz, and the phase lead of the eye movement against the turntable was 16.1° at 0.5 Hz and 4.88° at 2.5 Hz.

**Conclusions:**

To the best of our knowledge, this is the first report of this algorithm being used to calculate a 3D rotation vector of eye movement in mice using high-speed VOG. We developed a technique for analyzing the 3D rotation vector of eye movements in mice with a high-speed infrared CCD camera. We concluded that the technique is suitable for analyzing eye movements in mice. We also include a C++ source code that can calculate the 3D rotation vectors of the eye position from two-dimensional coordinates of the pupil and the iris freckle in the image to this article.

## Introduction

Eye movements are studied in many fields, including ophthalmology relating to neuro-otology relating to the vestibulo-ocular reflex (VOR), vision, neurology relating to the cerebellum’s role in movement, and psychology relating to attention. The VOR comprises the sensing of head movement and orientation by the vestibular system, which then makes compensatory adjustments in eye direction. The mouse is the most commonly used animal model in biomedical research because of recent advances in molecular genetic techniques. Therefore, measuring eye movements in mice is important for assessing multiple neural functions and the effects of genetic defects or therapeutic interventions on those functions that are of interest, including the vestibulo-ocular motor system, cerebellum motor control, and attention.

Two main methods are currently being used to analyze the eye movements of mice: the scleral search coil system [1] and video-oculography (VOG) [2–9]. The temporal resolution of the scleral search coil system was much greater than that of VOG as the sampling rate for VOG images was 30 Hz. Today, the sampling rate of VOG images has increased to more than 240 Hz [10]. Because this rate is high enough to analyze even saccadic eye movements, the temporal resolution of VOG is now sufficient for comprehensive assessment of eye movement, thereby avoiding problems associated with the use of coils.

## Materials and Methods

Here, we describe the method for analyzing movement of the turntable used in the study, our data acquisition and relevant analyses. We provide details on the algorithm used to analyze the three-dimensional (3D) rotation vector of mouse eye movements. Using these data, we calculated the VOR gain and phase in mice during rotation.

### Animals

Ten male C57BL/6J mice at 9–10 weeks of age and weighing 20–26 g were used in this study. The animals were purchased from Japan SLC Inc. (Hamamatsu, Japan). This study was carried out in strict accordance with the recommendations in the Guide for the Care and Use of Laboratory Animals of the National Institutes of Health. The Osaka University School of Medicine Animal Care and Use Committee approved the protocol of the study (Permit Numbers: 21-086-0, 27-043-000). All surgery was performed under anesthesia, and every effort was made to minimize animal suffering and reduce the number of animals used. After the experiments, we euthanized the animals by intraperitoneal injection of sodium pentobarbital (Nembutal; 200 mg/kg body weight).

### Surgical procedure

The mice were anesthetized with an intraperitoneal injection of a mixture of ketamine (100 mg/kg) and xylazine (10 mg/kg) in conjunction with local anesthesia (1% lidocaine). We made a small incision in the mouse’s head skin and fixed a small metal plate with a screw hole to the center of the skull using dental cement (Sun Medical, Shiga, Japan). After the surgery, the mice were isolated and closely observed for 48 h.

### Analyzing eye movement for calibration to reconstruct 3D coordinates of the eyeball

**Fig 1:**
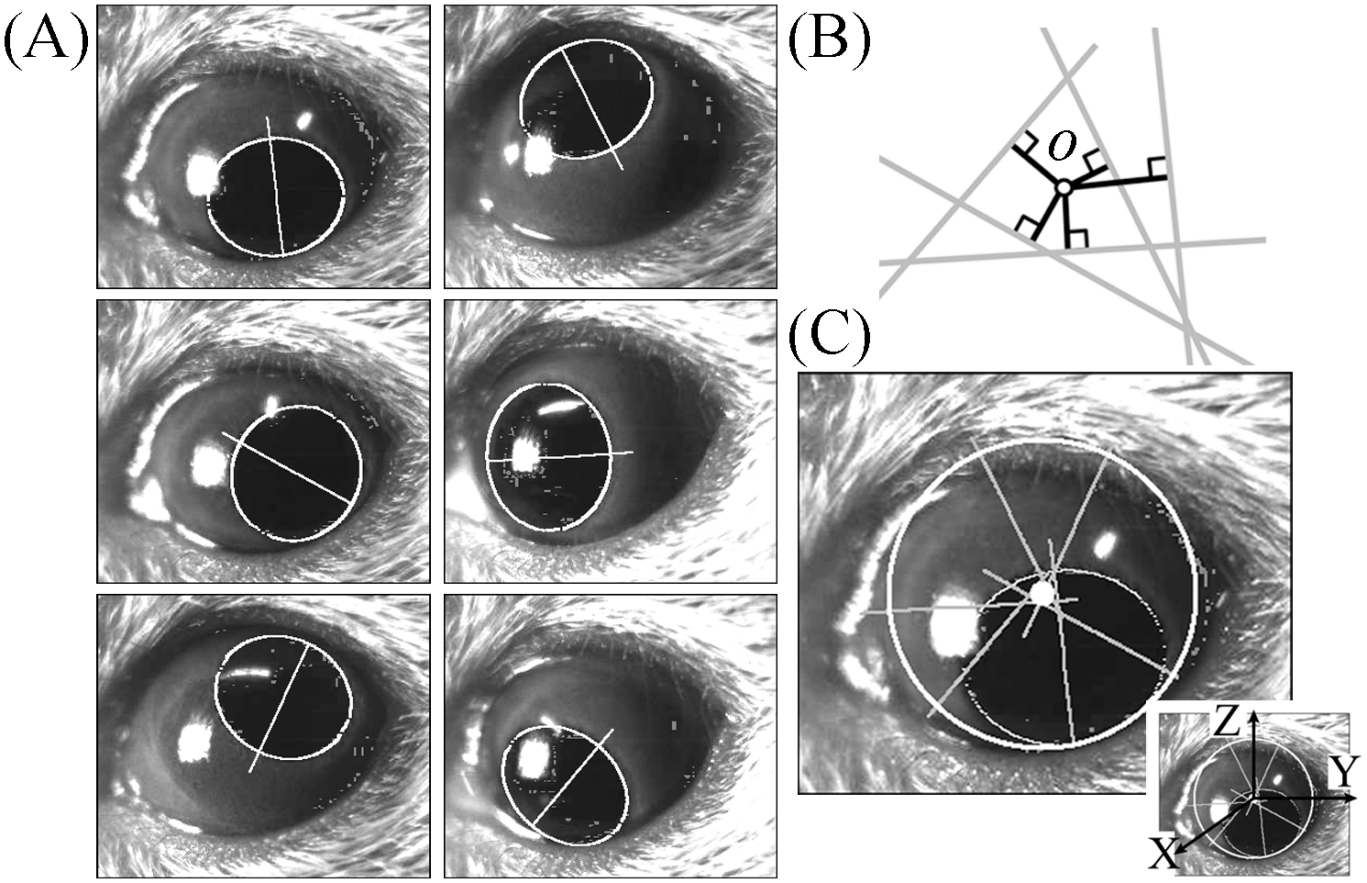
Calculation of the center of eye rotation in a two-dimensional image plane. (A) Pupil ellipses and the minor axis of the pupil ellipse obtained during circular eye movement induced by swirling the mouse around manually. (B) Point of intersection of multiple minor axes of a pupil ellipse. Ideally, multiple minor axes of a pupil ellipse would intersect at a single point, but they did not. The point of intersection of multiple minor axes of a pupil ellipse were determined as a point of the sum of the squares of the minimum distances between the point and the minor axes (black lines). (C) Center of eye rotation in the image calculated from the six minor axes shown in (A). The six minor axes shown in (A) are drawn here. The white small circle was the point where the sum of the squares of the distance between the point and the minor axis was minimum. After calculating the coordinates of the center of eye rotation in the image plane, a three-dimensional coordinate frame of XYZ was determined, as shown in the Inset.

Each mouse was placed in a plastic cylinder with a metal bar on a square (20 cm on each side) plastic board on a turntable (60 cm diameter). The metal plate on the mouse’s head was screwed onto the bar on the tube under inhalational anesthesia with 3% isoflurane. After awakening, the mouse on the plastic board was then swirled around manually, and the rotation-induced eye movements were recorded by a digital video camera. During this movement, the images of various ocular positions—abduction, adduction, supraduction, deorsumduction—were systematically recorded (Fig 1A). We obtained at least three images of each eye position.

### Fixation of mice to the turntable

**Fig 2:**
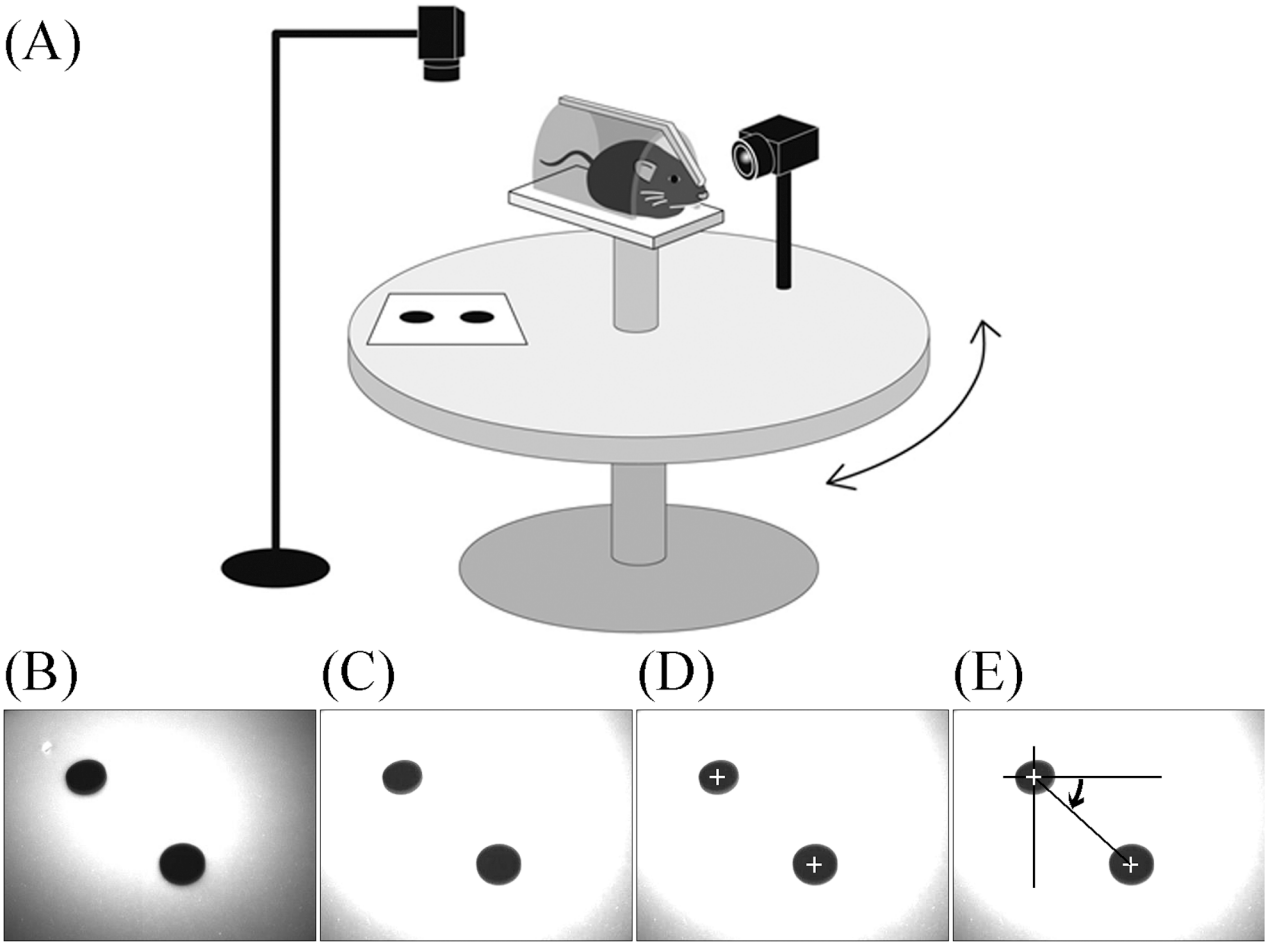
Schema for analyzing three-dimensional rotation vectors of eye movements during rotation in mice and the image-processing procedure for analyzing movement of the turntable. (A) Movements of the turntable and the eyes of the mice were recorded by two high-speed infrared CCD cameras. Images obtained by the two cameras were synchronized. (B) Original image of the markers on the turntable. (C) Same as that in (B) but in a contrast-enhanced image. (D) The center of gravity of the two markers was detected. (E) The movement of the line connecting the two centers of gravity of the markers was calculated to be equivalent to the movement of the turntable.

The mice were placed in a plastic cylinder with a metal bar at the center of a turntable and fixed to the metal bar so the mouse’s head was completely still during the rotations. In this case, however, the head was fixed 30° nose-down to align the lateral semicircular canals of the vestibular system with the horizontal plane [11] (Fig 2A).

### Analysis during rotation of the turntable

The turntable-associated eye movements were recorded using a high-resolution infrared camera recording system (sampling rate 240 Hz) (Sentech, Kanagawa, Japan). The acquisition of turntable images was synchronized with acquisition of the eye images using software (StreamPix; NorPix, Montreal, Canada). The custom-built metal plate, cylinder tube, turntable, camera holder, and all other custom equipment were made by Bio-medica (Osaka, Japan).

The two markers on the turntable were recorded from a position directly above the turntable, as shown in Fig 2A. The original image of the markers on the turntable is shown in Fig 2B. A contrast-enhanced image is shown in Fig 2C. The coordinates for the center of gravity of the two markers were extracted (Fig 2D). The movement of the turntable was measured off-line from the movement of the straight line passing through the two markers (Fig 2E).

### Eye movement recordings and image processing

**Fig 3:**
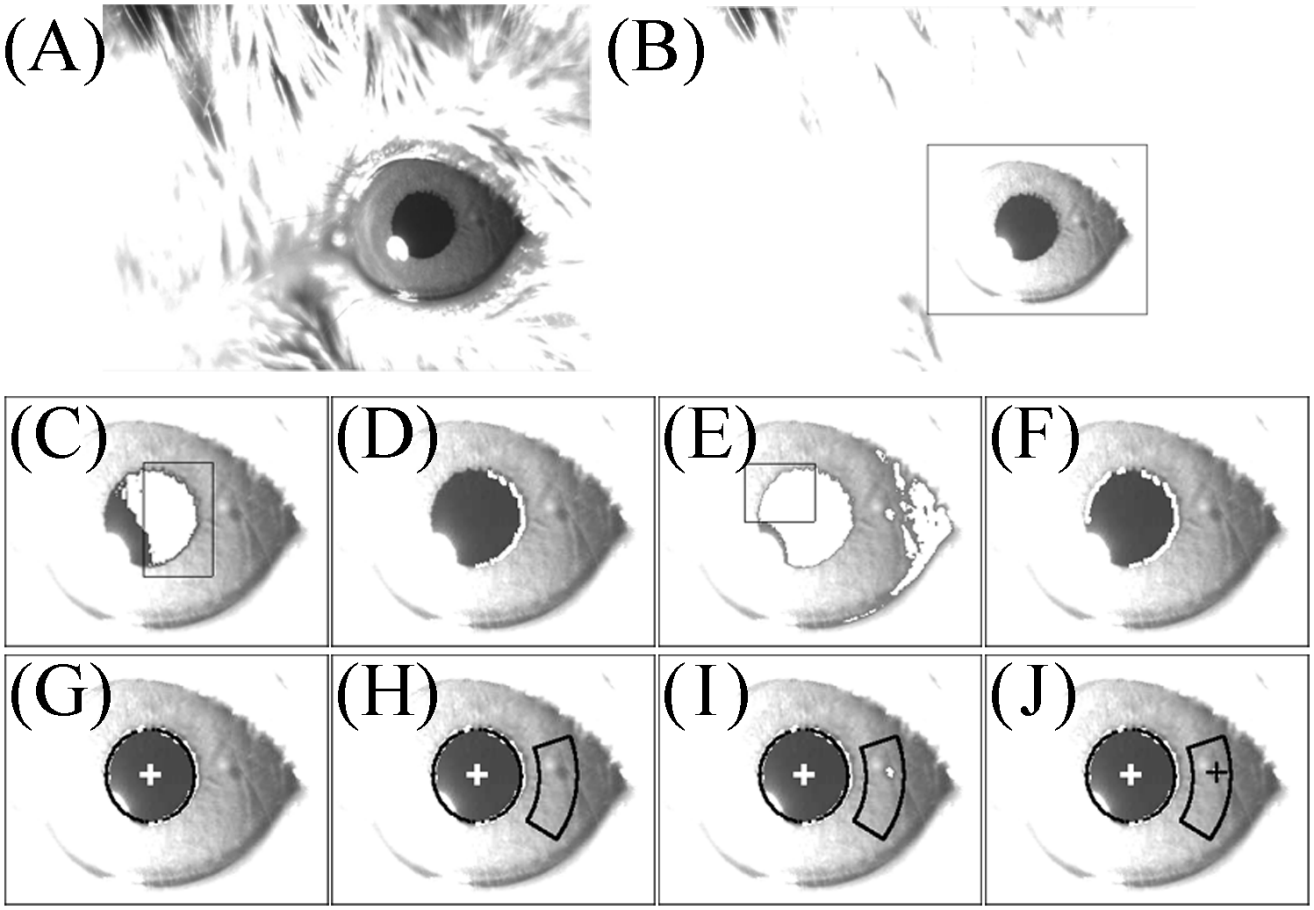
Image-processing procedure for detecting the center of the pupil ellipse and the center of gravity of an iris freckle in mouse A. (A) Original image of the left eye of mouse A. (B) Same eye as in (A) but in a contrast-enhanced image. (C) White areas are where the gray scale was less than the threshold value. Black rectangle shows the region of interest (ROI) for detecting the left edge of the pupil. (D) Left edge of the pupil was detected. (E) White areas are where the gray scale was less than the threshold value. Black rectangle shows the ROI for detecting the right edge of the pupil. (F) Right edge of the pupil was detected. (G) The edge of the pupil was approximated by the pupil ellipse and calculated at the center of the ellipse. (H) ROI for detecting an iris freckle was determined after calculating the pupil ellipse. The region was some distance from the center of the ellipse and had the same curvature as the ellipse. (I) In the region shown in (H), an iris freckle was detected with the gray scale less than the threshold value. (J) Coordinates of the iris freckle were calculated as the center of gravity of the white areas shown in (I).

The eye movements were recorded using the same high-resolution infrared camera recording system that was used to record the turntable movement. JPEG digital images of the eye movements were acquired using StreamPix software. The software for analyzing eye and turntable movement was written in Visual C++ packaged in Visual Studio 2013 (Microsoft, Edmond, WA, USA). The image-processing procedure, which is performed off-line, is shown in Fig 3. Fig 3A is the original grayscale image of the eye of mouse A. Fig 3B is a contrast-enhanced image of the same view. The area to be analyzed was determined from this image. The surrounding area in Fig 3B was used for the following process. Regions with a gray value that was at the threshold level or less were extracted (white area in Fig 3C), and the region of interest (ROI) was determined (area within the rectangle in Fig 3C). The left edge of the pupil was thus detected (Fig 3D). Regions with a gray value of another threshold or less were then extracted (white area in Fig 3E), and the ROI was determined (Fig 3E). Thus, the right edge of the pupil was detected (Fig 3F). These extracted edges of the pupil were approximated by the ellipse of the pupil (black curved line in Fig 3G), and the center of the pupil was determined (*yp zp*) (white cross in Fig 3G). The location of the white cross is the two-dimensional (2D) coordinate of the center of the pupil. The ROI (Fig 3H) was determined from the center of the pupil and the curvature of the pupil ellipse. The regions with a gray value at threshold or less were extracted (Fig 3I). The centroid of the extracted region was then determined (*yi zi*) (black cross in Fig 3J). The location of the black cross was the 2D coordinate of an iris freckle.

### Assumptions for rotation vector analysis

The principle for constructing the 3D coordinates of the center of the pupil or an iris freckle is based on the following four assumptions: (1) the eye rotates about a point; (2) the pupil edge is a circle, and the edge and iris freckles exist on the same plane; (3) the distance from the center of eye rotation to the pupil circle remains unchanged despite eye rotation; (4) images of the eye obtained by a digital camera are projected onto a plane that is perpendicular to the camera axis [12].

### Calculation of the coordinate of the center of eye rotation

Subjected to the four assumptions described above, the 2D coordinate of the center of eye rotation *o* is determined as the intersection of the extensions of the minor axes of the pupil ellipse because *o* is on an extension of the minor axis of the pupil ellipse (Fig 1). Although, theoretically, all minor axes intersect each other at one point *o*, in actuality they do not. The intersection of all minor axes (*yc zc*) is determined as the point where the averaged square of the distance between the point and the extensions of the minor axes of the pupil ellipses is minimum (Fig 1B and 1C). Thus, after calculating the coordinate of the center of eye rotation, the 3D coordinate frame XYZ is determined (inset, Fig 1C). The eye position of that coordinate of the center of the pupil that is nearest to the coordinate (*yc zc*) is set as the reference position.

### Calculation of the radius of rotation of the pupil center

**Fig 4:**
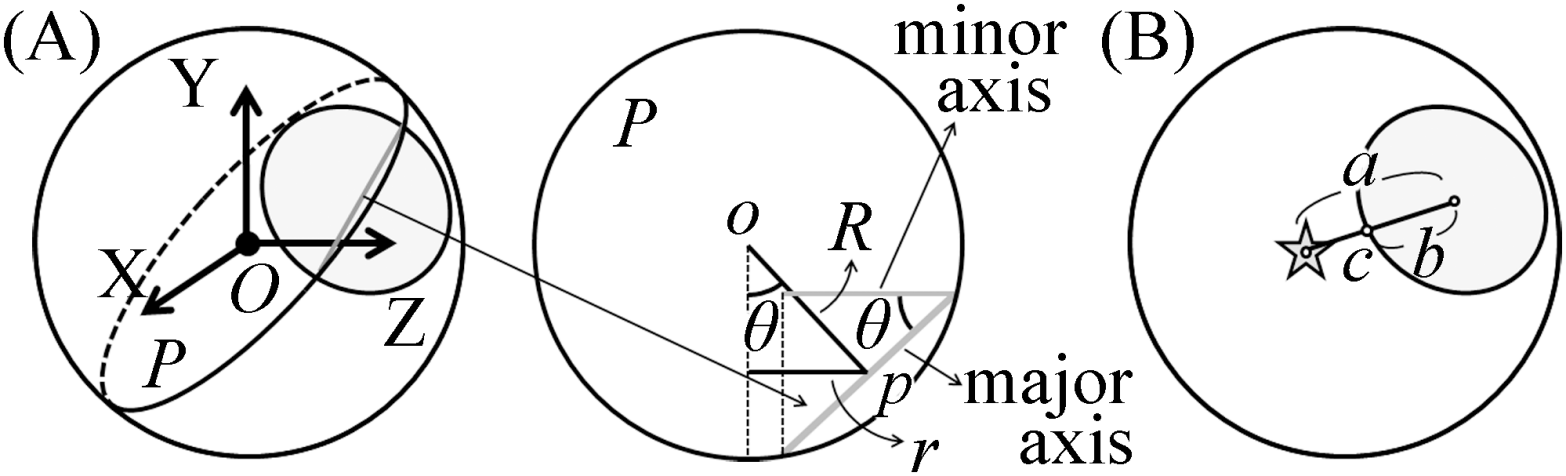
Calculation of the length of the radius of rotation of the center of the pupil and the radius of rotation of an iris freckle. (A) Calculation of the length of the radius of rotation of the pupil center, *R*. Eqs 1–3 apply when the eye rotates *θ* from the eye position during frontal vision in the plane *P*, which includes the center of eye rotation and the diameter that corresponds to the minor axis of the pupil ellipse of the image plane (gray line, left figure), where *r* is the length between *o* and the center of the pupil ellipse *p*. (B) Next, we calculate the length of the radius of rotation of an iris freckle. When an iris freckle is on the same plane as the pupil edge, the radius of rotation, *R’*, is calculated by Eq 4, in which *a* is the distance between the center of the pupil ellipse and the center of gravity of an iris freckle in the image, *b* is the distance between the center of the pupil ellipse and point *c* in the image, point *c* is the point of intersection of the edge of the pupil ellipse and the line connecting of the center of the pupil ellipse and the center of gravity of an iris freckle in the image.

After calculating the coordinate of the center of eye rotation, the radius of rotation of the center of the pupil (*R*) can be calculated. When the eye rotates *θ* from its position during frontal vision (Fig 4A), in plane *P* (which includes the center of eye rotation and the diameter that corresponds to the minor axis of the pupil ellipse of the image plane) (Fig 4A), the following formulas are correct:

$$\text{cos}\theta =\frac{\text{length}\;\text{of}\;\text{minor}\;\text{axis}\;\text{in}\;\text{the}\;\text{pupil}\;\text{ellipse}\;\text{in}\;\text{the}\;\text{image}}{\text{length}\;\text{of}\;\text{major}\;\text{axis}\;\text{in}\;\text{the}\;\text{pupil}\;\text{ellipse}\;\text{in}\;\text{the}\;\text{image}}$$ (1)

$$\text{sin}\theta =\frac{r}{R}$$ (2)

where *r* is the distance between *o* and the center of the pupil ellipse *p* in the image.

Because cos^2^*θ* + sin^2^*θ* = 1,

$$R=\frac{r}{\sqrt{1-{\left(\frac{\text{length}\;\text{of}\;\text{minor}\;\text{axis}\;\text{in}\;\text{the}\;\text{pupil}\;\text{ellipse}\;\text{in}\;\text{the}\;\text{image}}{\text{length}\;\text{of}\;\text{major}\;\text{axis}\;\text{in}\;\text{the}\;\text{pupil}\;\text{ellipse}\;\text{in}\;\text{the}\;\text{image}}\right)}^{2}}}$$ (3)

Using the above formula, we can obtain the *R* for each pupil ellipse. The average value of the *R*s is adopted.

### Calculation of the radius of rotation of an iris freckle

When an iris freckle exists on the same plane with the pupil edge, the following formula is true (Fig 4B):

$$R\prime^{2}=R^{2}+{\left(\;\frac{a}{b}\cdot \frac{\;\text{length}\;\text{of}\;\text{major}\;\text{axis}\;\text{of}\;\text{pupil}\;\text{ellipse}\;\text{in}\;\text{the}\;\text{image}}{2}\;\right)}^{2}$$

$$R\prime =\sqrt{R^{2}+{\left(\;\frac{a}{b}\cdot \frac{\text{length}\;\text{of}\;\text{major}\;\text{axis}\;\text{of}\;\text{pupil}\;\text{ellipse}\;\text{in}\;\text{the}\;\text{image}}{2}\;\right)}^{2}}\;$$ (4)

where *R'* is the radius of rotation of an iris freckle, *a* is the length between the center of the pupil ellipse and the center of gravity of an iris freckle (Fig 4B), *b* is the distance between the center of the pupil ellipse and point *c* in the image, and point *c* is the point of intersection of the edge of the pupil ellipse and the line connecting the center of the pupil ellipse and the center of gravity of an iris freckle (Fig 4B). The average value of the *R′*s is adopted.

### Calculation of the 3D coordinates of the pupil center and an iris freckle

The space coordinates for calculating the 3D coordinates of the pupil center and an iris freckle were defined as follows: The X axis is perpendicular to the image plane (positive forward); the Y axis is parallel to the horizontal axis of the image plane (positive right from the examiner’s point of view); and the Z axis is parallel to the vertical axis of the image plane (positive upward) (inset, Fig 1C). The X, Y, and Z components mainly reflect roll, pitch, and yaw components, respectively. The direction of rotation was described according to the point of view of the mice. The 3D coordinate of the center of eye rotation *O* (Fig 4A) is (0 0 0).

The 3D coordinate of the center of the pupil is

$$\left(\sqrt{R^{2}-{\left(yp-yc\right)}^{2}-{\left(zp-zc\right)}^{2}}\;yp-yc\;zp-zc\right).$$

The 3D coordinate of an iris freckle is

$$\left(\sqrt{R{’}^{2}-{\left(yi-yc\right)}^{2}-{\left(zi-zc\right)}^{2}}\;yi-yc\;zi-zc\right).$$

### Calculation of the rotation vector of eye position and velocity

First, arrange the (1) 3D coordinate of the center of the pupil (*xpr ypr zpr*), the 3D coordinate of the iris freckle (*xir yir zir*), and the cross product of (*xpr ypr zpr*) and (*xir yir zir*)—(*xcr ycr zcr*) when the eye is in the reference position. (2) Arrange the 3D coordinate of the center of the pupil (*xpa ypa zpa*), the 3D coordinate of the iris freckle (*xia yia zia*), and the cross product of (*xpa ypa zpa*) and (*xia yia zia*)—(*xca yca zca*) when the eye is in a given eye position. Then, the matrix *M*, which represents the given eye position, is calculated as follows:

$$M\left(\begin{array}{ccc} xpr & xir & xcr\\ ypr & yir & ycr\\ zpr & zir & zcr \end{array}\right)\;=\;\left(\begin{array}{ccc} xpa & xia & xca\\ ypa & yia & yca\\ zpa & zia & zca \end{array}\right)$$

$$M\;=\;\left(\begin{array}{ccc} xpa & xia & xca\\ ypa & yia & yca\\ zpa & zia & zca \end{array}\right){\left(\begin{array}{ccc} xpr & xir & xcr\\ ypr & yir & ycr\\ zpr & zir & zcr \end{array}\right)}^{-1}\;=\;\left(\begin{array}{ccc} R11 & R12 & R13\\ R21 & R22 & R23\\ R31 & R32 & R33 \end{array}\right).$$

The rotation vector **r**, which represents the given eye position, is calculated from matrix *M* [13]:

$$r\;=\;\frac{1}{1+\left(R11+R22+R33\right)}*\left(\begin{array}{c} R32-R23\\ R13-R31\\ R21-R12 \end{array}\right).$$

The rotation vector of eye velocity **ω** is also calculated [13]:

$$\omega \;=\;2*\frac{\frac{\text{d}r}{\text{dt}}+\text{r}\times \frac{\text{d}r}{\text{dt}}}{1+r^{2}}.$$

The source code of the program (S1 Source) that can calculate the 3D rotation vector of eye position **r** from the 2D coordinates of center of pupil (*yp*-*yc zp*-*zc*) and iris freckle (*yi*-*yc zi*-*zc*) is attached to the article.

### Analyzing eye movement in mice during rotation

The eye movements of the mice and the movement of the turntable were recorded simultaneously while the turntable was being rotated manually in darkness, with a metronome set at 0.5 Hz and 2.5 Hz. We collected eye movement data during rotation in three mice. Before rotation, the pupils in two mice (mice A and B) were contracted with an ophthalmic solution (1% pilocarpine hydrochloride; Nippon Tenganyaku Kenkyusho, Nagoya, Japan). The pupil in the other mouse (mouse C) was dilated because the mouse was not given a miotic drug. The recorded images of eyes and turntable were then analyzed as described above. The angular velocity data of the turntable were approximated by the formula, *vt*·sin(2π*f*t+*pt*). Next, using the frequency, *f*, which was calculated from the turntable data, the angular velocity data of the eyes were approximated by the formula, *ve*·sin(2π*f*t+*pe*).

## Results

**Fig 5:**
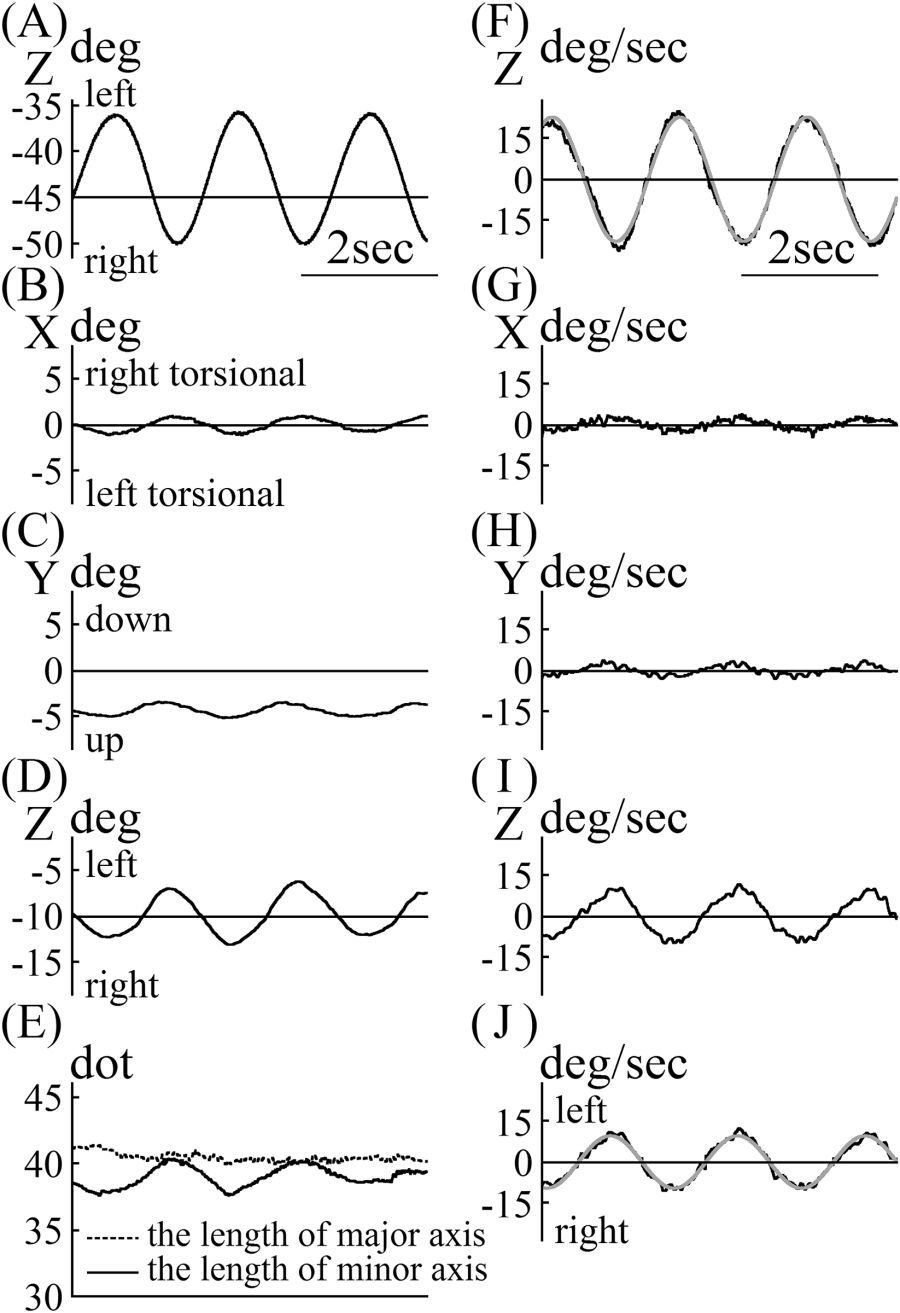
Vestibulo-ocular reflex (VOR) data for mouse A. (A) Data for the position of the turntable during rotation. (B) X component of the rotation vector of eye position during rotation. (C) Y component of the rotation vector of eye position during rotation. (D) Z component of the rotation vector of eye position during rotation. The Z component was in phase with the X and Y components, but it was 180° out of phase with the turntable shown in (A). (E) Changes in the length of the minor and major axes of the pupil ellipse during rotation. The length of the major axis of the pupil ellipse was almost constant during rotation. The length of the minor axis of pupil ellipse changed in phase with the Z component phase, as shown in (D). (F) Velocity data of the turntable during rotation. (G) X component of the rotation vector of eye velocity during rotation. The length of the minor axis of the pupil ellipse changed in phase with the Z component change, as shown in (D). (F) Velocity data for the turntable during rotation. (G) X component of rotation vectors of eye velocity during rotation. (H) Y component of rotation vectors of eye velocity during rotation. (I) Z component was the main component of the rotation vectors of eye movement. X and Y component values were low. Z component was 180° and was out of phase with the turntable, as shown in (F). (J) Angular velocity of eye rotation around the axis of rotation. As the value of the rotation vectors of eye velocity were always positive, we let the sign of angular velocity of eye rotation around the axis of rotation coincide with the Z component of the rotation vector of eye velocity. We calculated the gain and phase of VOR using these data.

The angular position data of the turntable and the eye of mouse A, whose pupil was strongly contracted with a miotic drug, are shown in Fig 5. As shown in Fig 3, the entire pupil edge could be seen. Mouse A was rotated with a metronome set at 0.5 Hz. The main component of eye movement was the Z component. The phases of the X and Y eye movement components were the same as that of the Z component. The Z component phase was out of phase with that of movement of the turntable. The lengths of the major axes of the pupil ellipse in the images were almost constant, regardless of the eye position (Fig 5E). Changes in the length of the minor axis of the pupil ellipse in the images were synchronized with the change in eye position (Fig 5D and 5E). Angular velocity data for the turntable are shown in Fig 5F. The angular velocity was approximated by the following formula:

23.0sin(2π(0.53)t+1.073).

Z was also the main component in eye angular velocity. The X and Y components of that phase were the same that of the Z component but were weak. We calculated the eye angular velocity around the axis of eye rotation, |**Ve**|.

$$\left|Ve\right|\;=\;\sqrt{{\left(\text{X}\;\text{component}\;\text{of}\;Ve\right)}^{2}+{\left(\text{Y}\;\text{component}\;\text{of}\;Ve\right)}^{2}+{\left(\text{Z}\;\text{component}\;\text{of}\;Ve\right)}^{2}}$$

As the sign of |**Ve**| was always plus, the sign of |**Ve**| coincided with the sign of the Z component of **Ve**. In Fig 5J, the abscissa is the time axis, and the ordinate represents the eye angular velocity around the axis of eye rotation. The eye angular velocity around the axis was approximated by the following formula using a frequency of 0.53 Hz, which was obtained by approximating the angular velocity of the turntable:

9.7sin(2π(0.53)t−1.787).

If the eye movement compensates the movement of the turntable completely, the formula for eye angular velocity should be

23.0 sin(2π(0.53)t+1.073−π).

Comparing actual eye angular velocity with this ideal formula for eye angular velocity, the gain was calculated as 0.42 (9.7 / 23.0), and the phase was calculated as 0.282 radian (16.1°) [−1.787 − (1.073 − π)]. The phase 16.1° means that the eye angular velocity led the angular velocity of the turntable by 16.1°.

**Fig 6:**
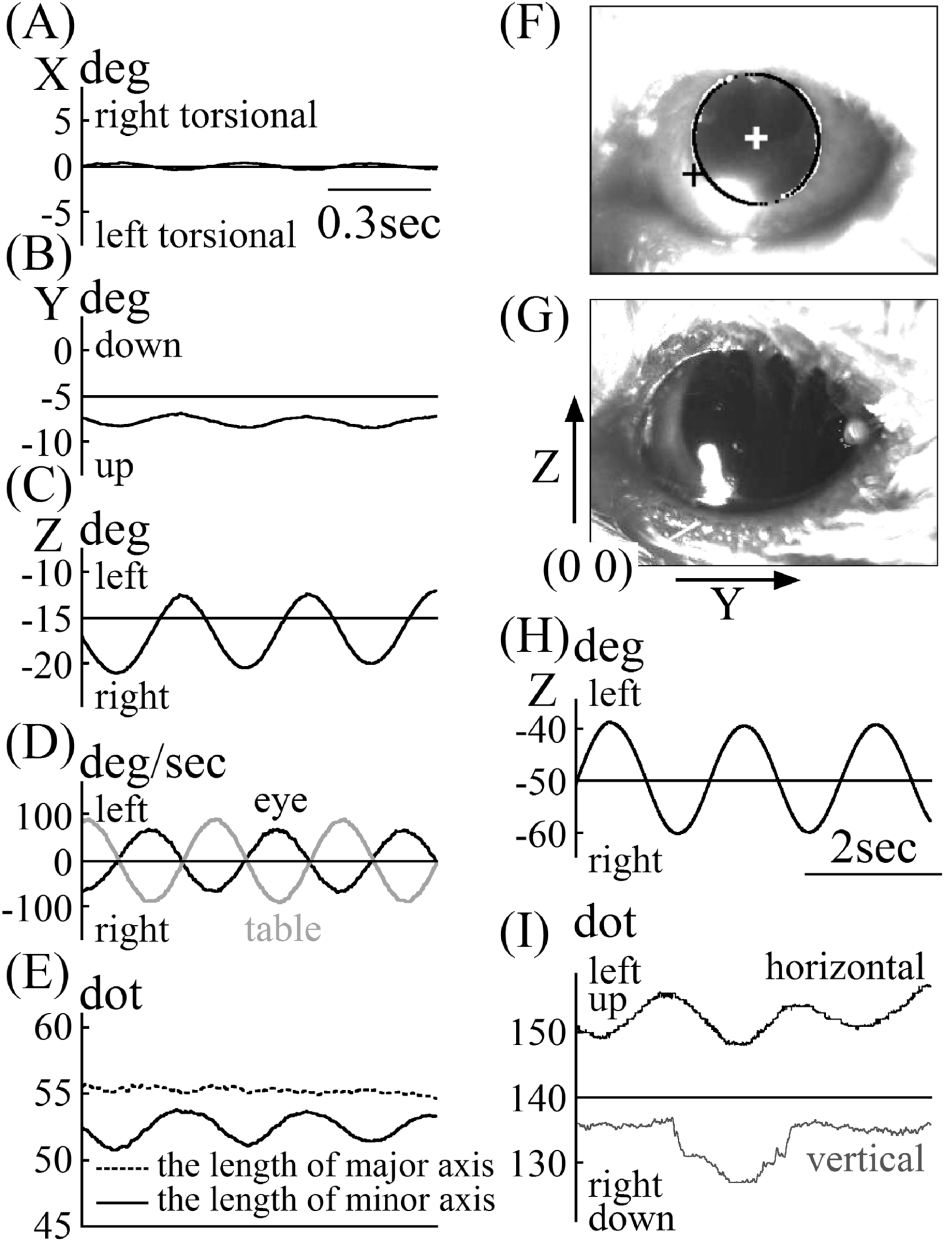
VOR data for mice B and C. A, X component of the rotation vector of eye position during rotation in mouse B. B, Y component of the rotation vector of eye position during rotation in mouse B. C, Z component of the rotation vector of eye position during rotation in mouse B. D, Angular velocity of eye rotation around the axis of rotation in mouse B and velocity data of the turntable during rotation. The gain of VOR was 0.74 [(65.7°/sec) / (88.5°/sec)] and the phase of VOR was 4.88°. E, Changes in the length of the minor and major axes of the pupil ellipse during rotation in mouse B. The length of the major axis of the pupil ellipse was almost constant during rotation. The length of the minor axis of pupil ellipse changed in phase with the Z component phase, as shown in (C). F, Contrast-enhanced image of the eye of mouse B. G, Contrast-enhanced image of the eye of mouse C. H, Data for the position of the turntable during mouse C was rotated. I, The movement of the coordinate of center of pupil of mouse C in two-dimensional images. The unit of this graph was “dots,” not “degrees” because the iris freckle could not be detected, and the rotational angle could not be analyzed.

The angular position and velocity data of mouse B’s eye, whose pupil was slightly contracted with a miotic drug, are shown in Fig 6A–6D. Although as shown in Fig 6F the upper side of the pupil slightly touched the upper eyelid, almost the entire pupil edge could be narrowly seen. Mouse B was rotated with a metronome set at 2.5Hz. The data quality of the eye position and velocity for mouse B was equivalent with that in mouse A. The gain of VOR in mouse B was 0.74 [(65.7°/sec) / (88.5°/sec)], and the phase of VOR was 4.88° (Fig 6D). As with mouse A, the lengths of the major axes of the pupil ellipse in the images were almost constant, regardless of the eye position (Fig 6E). Also, changes in the length of the minor axis of the pupil ellipse in the images were synchronized with the changes in eye position (Fig 6C and 6E). The length of the major axis of pupil ellipse for mouse B was 15 dots greater than that of mouse A (Fig 5E vs. Fig 6E).

The eye position data in the images of mouse C—whose pupil was dilated because the mouse had not been given a miotic drug and had been rotated in darkness—and the turntable data are shown in Fig 6H and 6I. As shown in Fig 6G, when the pupil was dilated, the iris freckle could not be seen. Mouse C was rotated with a metronome set at 0.5 Hz. To analyze the eye’s angular position using this method, information regarding the coordinate of the iris freckle is essential. Therefore, we could not analyze the angular eye position of mouse C. We show only the movement of the coordinate of the center of the pupil in the 2D image in Fig 6I. Although the coordinate of the horizontal direction of the center of the pupil in mouse C (Y component, *yp*) was not smooth, it followed a sine curve (Fig 6I) synchronized with the movement of the turntable (Fig 6H). The coordinate of the vertical direction of the center of the pupil in mouse C (Z component, *zp*) was not smooth and did not follow a sine curve (Fig 6I), although logically it should follow a sine curve, similar to the coordinate in the horizontal direction. The results of the coordinates of the horizontal and vertical directions of the center of the pupil in mouse C mean that the coordinate of the center of the pupil of mice similar mouse C, whose pupil was not contracted and whose pupil edge could be seen only partially, cannot be extracted well.

## Discussion

We developed a new technique for analyzing the rotation vectors of eye movement in three dimensions in mice using a high-speed infrared CCD camera. We were able to determine the VOR gain and phase in the mice during rotation using this system.

With contrast enhancement (Fig 3A and 3B), it was easy to detect the edge of the pupil and iris freckles. For detecting the the pupil’s edge, the multi-level image was binarized the same way as with another VOG system [14]. The difference between the two VOG techniques is that we used different thresholds to detect the right and left edges of the pupil by binarization. To the naked eye, it appears that there is no difference in brightness between the right and left sides of the pupil because the brightness varies gradually but continuously. Because of the influence of the position of the infrared light source, however, the brightness gradient between the right and left sides of the pupil are apparent on the images. The right side of the pupil shown in Fig 3A is brighter than of the left side. We also changed the ROI between detecting the right edge and the left edge of the pupil (Fig 3C and 3E). As a result, it was possible to detect the edges of the pupil accurately (Fig 3F). The pupil edges were then approximated by the pupil’s ellipse, and the coordinate for the center of the pupil ellipse was found (Fig 3G). An ROI that had the same curve as that of the pupil ellipse was set at a fixed distance away from the center of the pupil ellipse (Fig 3H), and a contrast-enhanced iris freckle was detected (Fig 3I). Finally, the center of gravity of the iris freckle was detected (Fig 3J).

Circular eye movement is necessary to find the coordinates of the center and the radius of eye rotation. It is easy to obtain images during circular eye movement in humans as they are simply asked to follow targets arranged on a circle [12]. In mice, we obtained the images during rotation-induced circular eye movement by swirling the mice around manually (Fig 1A). The coordinates for the center and radius of eye rotation were calculated using the minor axes of pupil ellipses and the ratio of the length of the minor axis to the length of the major axis (Figs 1B and 1C and 4A). When calculating them in humans we use 200 images at a 30-Hz sampling rate [12], whereas in mice we used 1600 images with a 240-Hz sampling rate. While obtaining those 1600 images, the mice rotated their eyes about three times. In Fig 1C, the circle at the center of the eye was the center of rotation, and the radius was that of eye rotation. This circle was smaller than the eyeball, which makes sense because the radius we calculated was the radius of the rotation of the center of pupil, not the radius of the eyeball. After calculating the radius, we calculated the radius of rotation of the iris freckle (Fig 4B). Using these radii, we calculated the 3D coordinates of the center of the pupil and the iris freckle.

Any eye position is represented as relative to the reference eye position [13]. From the 3D coordinates of the center of the pupil and the iris freckle when the eye is at a reference position, the rotation vector of the eye position **r** can be calculated. The value of |**r**| is tan*θ*/2, not the Euler angle. Because it is easier to understand eye rotation when using the Euler angle than when using tan*θ*/2, we translated the rotation vector **r** to axis angle **a** [15]. Axis angle **a** is calculated using the following formula:

$$\text{a}=\frac{2{\text{tan}}^{-1}\left|\text{r}\right|}{\left|\text{r}\right|}\text{r}.$$

Using StreamPix software, we could synchronize the images recorded by two cameras so images of the movement of the turntable were recorded simultaneously with the eye movements of the mice. The rotation angle of the turntable was calculated from the movement of two markers fixed on the turntable (Fig 2). In darkness, the turntable was rotated manually with a metronome that was set at 0.5 Hz, inducing the VOR in the mice. We manually rotated the turntable sinusoidally, as shown in Fig 5A. The rotation was approximated using the formula 23.0 sin(2π(0.53)t+1.073). The approximated frequency of rotation was 0.53 Hz, although the metronome was set at 0.50 Hz. For approximation of eye angular velocity, however, 0.53 Hz was used. The VOR of mice was induced during rotation. Because the mice did not have saccadic eye movements, we did not have to go through the process of removing them when calculating the slow-phase eye velocity. If the process had become necessary, however, we had developed a program to address that problem [16]. Eye movement during VOR had three components, X, Y, and Z, with the main component being Z (Fig 5). To assess the VOR using only one parameter, not three, we used the eye angular velocity around the axis of eye rotation (Fig 5J) [17]. The angular eye velocity was approximated by 9.7sin(2π(0.53)t−1.787). The gain of VOR was 0.42 (9.7 / 23.0), and the phase was 16.1° (0.282 rad): −1.787 − (1.073 − π). These values were the same as those that had already been reported [18].

When we find the coordinate of the center of the pupil ellipse of mice, we use the coordinates of the edges of the pupil ellipse in 2D images; thus, the most accurate coordinate of the center of the pupil is detected when all edges of the pupil ellipse can be detected. Moreover, when the size of the pupil is small, it is easy to detect all edges of the pupil ellipse; for this reason, we used a miotic drug to contract the pupil of mouse A (Fig 3). When the pupil was contracted, the iris freckle could be seen clearly (Fig 3). When we can detect the coordinates of the center of the pupil and iris freckle accurately, the system we have described works properly. We examined whether this system worked well when the pupil was large. Although mouse B was given a miotic drug, its pupil contraction was weaker than that of mouse A (Fig 3 vs. Fig 6F) and the length of the pupil ellipse of mouse B was 15 dots greater than that of mouse A (Fig 5E vs. Fig 6E). As shown in Fig 6F, part of the pupil edge of mouse B touched the upper eyelid. Because the entire pupil edge and iris freckle could be narrowly detected (Fig 6F), the 3D eye position and eye velocity around the axis of rotation in mouse B could be analyzed with the same quality as that in mouse A (Fig 6A–6D). The gain of VOR in mouse B was 0.74 [(65.7°/sec) / (88.5°/sec)], and the phase of VOR in mouse B was 4.88° at 2.5 Hz (Fig 6D). We also examined whether the system worked when the pupil was dilated as in mouse C. The pupil of mouse C was dilated because mouse C had not been given a miotic drug and was rotated in darkness. As shown in Fig 3G, only the right edge of the pupil ellipse could be seen (not the iris freckle). Because only the right edge of the pupil was used when the edges of the pupil were approximated by the ellipse, the movement of the coordinate of the center of the pupil ellipse could not be detected accurately on 2D images (Fig 6I). Because both accurate coordinates of center of pupil and iris freckle are essential, this system cannot work when murine pupils are dilated. When the length of the major axis of the pupil ellipse was less than that in mouse B, we could analyze the 3D rotation vector of eye movement by the system. When using this system, the pupil must be contracted using a miotic drug or by being exposed to light.

The response of the VOR changed with stimulus frequency, having a higher gain and smaller phase lead when the stimulus frequency was increased [8]. In this study, when the frequency of rotation was increased from 0.5 Hz to 2.5 Hz, the VOR gain increased from 0.53 to 0.74, and the VOR phase decreased from 16.1° to 4.88° (Fig 5F and 5J vs. Fig 6D). These results mean that the gain and phase of VOR could be analyzed properly in mice. In our previous studies, the accuracy of this system was verified using an eye movement simulator [12] and by comparing the results with those obtained using a scleral search coil system in humans [19].

## Conclusion

We developed a new technique for analyzing 3D rotation vectors of eye movements in mice using a high-speed infrared CCD camera. We concluded that the technique is suitable for analyzing eye movements in mice.

## Supporting Information

S1 DataSimulated 2D data for the center of the pupil.Simulated 2D data for the center of the pupil when the eye is rotated from −30° to +30° for each degree around the axis $\left(1/\sqrt{14}\;2/\sqrt{14}\;3/\sqrt{14}\right)$. In the file the length of the radius is shown in the first line, the coordinate when the eye is at a reference position is shown in the second line, and each 2D coordinate (*yp*-*yc zp*-*zc*) is shown in the following lines.(CSV)

S2 DataSimulated 2D data for the iris freckle.Simulated 2D data for the iris freckle when the eye is rotated from −30° to +30° for each degree around the axis $\left(1/\sqrt{14}\;2/\sqrt{14}\;3/\sqrt{14}\right)$. In the file the length of the radius is shown in the first line, the coordinate when the eye is at a reference position is shown in the second line, and each 2D coordinate (*yi*-*yc zi*-*zc*) is shown in the following lines.(CSV)

S3 Data 3D rotation vector file. When S1 and S2 Data are analyzed by S1 Source, we can obtain the 3D rotation vector file.
(CSV)

S1 Source The source code of the program. The source code of the program that can analyze 3D rotation vectors of eye positions from 2D data of the center of the pupil and iris freckle.
(DOCX)
