# Supplementary material for: High-Speed Video-Oculography for Measuring Three-Dimensional Rotation Vectors of Eye Movements in Mice
Source: PLoS One. 2016 Mar 29;11(3):e0152307. doi: 10.1371/journal.pone.0152307 (PMC4811530; doi:10.1371/journal.pone.0152307)
Supplement: S1 Source — The source code of the program that can analyze 3D rotation vectors of eye positions from 2D data of the center of the pupil and iris freckle. (DOCX) [file pone.0152307.s004.docx]

#include "stdafx.h"

#include "math.h"

String ^ pupil;

String ^ iris;

String ^ vector;

int i,j;

double xpr,ypr,zpr;

double xir,yir,zir;

double xcr,ycr,zcr;

double detA,rv;

double xpa[10000],ypa[10000],zpa[10000];

double xia[10000],yia[10000],zia[10000];

double xca[10000],yca[10000],zca[10000];

double r11,r12,r13,r21,r22,r23,r31,r32,r33;

double rvx,rvy,rvz;

double radius;

pupil = "S2_pupil.csv";

iris = "S3_iris.csv";

vector = "S4_vector.csv";

String^ strDelimiter =",";

array<Char>^ delimiter = strDelimiter->ToCharArray();

array<String^>^ strData;

String^ strLine;

Boolean fileExists =System::IO::File::Exists(pupil);

if(fileExists)

{

System::IO::StreamReader^ sr = gcnew System::IO::StreamReader(pupil, System::Text::Encoding::Default);

strLine = sr->ReadLine();

strData = strLine->Split(delimiter);

radius=double::Parse(strData[1]);

i=0;

while(sr->Peek()>=0)

{strLine = sr->ReadLine();

strData = strLine->Split(delimiter);

ypa[i]=double::Parse(strData[0]);

zpa[i]=double::Parse(strData[1]);

xpa[i]=sqrt((double)(radius*radius-ypa[i]*ypa[i]-zpa[i]*zpa[i]));

i=i+1;

}

sr->Close();

}

fileExists =System::IO::File::Exists(iris);

if(fileExists)

{

System::IO::StreamReader^ sr = gcnew System::IO::StreamReader(iris, System::Text::Encoding::Default);

strLine = sr->ReadLine();

strData = strLine->Split(delimiter);

radius=double::Parse(strData[1]);

for(j=0;j<i;j++)

{strLine = sr->ReadLine();

strData = strLine->Split(delimiter);

yia[j]=double::Parse(strData[0]);

zia[j]=double::Parse(strData[1]);

xia[j]=sqrt((double)(radius*radius-yia[j]*yia[j]-zia[j]*zia[j]));

}

sr->Close();

}

for(j=0;j<i;j++)

{

xca[j]=ypa[j]*zia[j]-zpa[j]*yia[j];

yca[j]=zpa[j]*xia[j]-xpa[j]*zia[j];

zca[j]=xpa[j]*yia[j]-ypa[j]*xia[j];

}

detA=xpa[0]*yia[0]*zca[0]+ypa[0]*zia[0]*xca[0]+zpa[0]*xia[0]*yca[0]-xpa[0]*yca[0]*zia[0]-zpa[0]*yia[0]*xca[0]-ypa[0]*xia[0]*zca[0];

xpr=yia[0]*zca[0]-yca[0]*zia[0];

ypr=yca[0]*zpa[0]-ypa[0]*zca[0];

zpr=ypa[0]*zia[0]-yia[0]*zpa[0];

xir=xca[0]*zia[0]-xia[0]*zca[0];

yir=xpa[0]*zca[0]-xca[0]*zpa[0];

zir=xia[0]*zpa[0]-xpa[0]*zia[0];

xcr=xia[0]*yca[0]-xca[0]*yia[0];

ycr=xca[0]*ypa[0]-xpa[0]*yca[0];

zcr=xpa[0]*yia[0]-xia[0]*ypa[0];

xpr=xpr/detA; ypr=ypr/detA; zpr=zpr/detA;

xir=xir/detA; yir=yir/detA; zir=zir/detA;

xcr=xcr/detA; ycr=ycr/detA; zcr=zcr/detA;

System::IO::StreamWriter^ sw=gcnew System::IO::StreamWriter(vector,false,System::Text::Encoding::Default);

String^ stpData = "";

for(j=1;j<i;j++)

{

r11=xpa[j]*xpr+xia[j]*ypr+xca[j]*zpr;

r12=xpa[j]*xir+xia[j]*yir+xca[j]*zir;

r13=xpa[j]*xcr+xia[j]*ycr+xca[j]*zcr;

r21=ypa[j]*xpr+yia[j]*ypr+yca[j]*zpr;

r22=ypa[j]*xir+yia[j]*yir+yca[j]*zir;

r23=ypa[j]*xcr+yia[j]*ycr+yca[j]*zcr;

r31=zpa[j]*xpr+zia[j]*ypr+zca[j]*zpr;

r32=zpa[j]*xir+zia[j]*yir+zca[j]*zir;

r33=zpa[j]*xcr+zia[j]*ycr+zca[j]*zcr;

rv=1.0+r11+r22+r33;

rvx=(r32-r23)/rv;

rvy=(r13-r31)/rv;

rvz=(r21-r12)/rv;

stpData = rvx.ToString()+","+rvy.ToString()+","+rvz.ToString();

sw->WriteLine(stpData);

}

sw->Close();

this->Close();
